# Implementation of elementary school physical education quantity and quality law through school district audit, feedback, and coaching

**DOI:** 10.1186/s12966-023-01479-1

**Published:** 2023-06-29

**Authors:** Hannah R. Thompson, Kristine A. Madsen, Maya Zamek, Thomas L. McKenzie, David A. Dzewaltowski

**Affiliations:** 1 School of Public Health, Community Health Sciences, UC Berkeley, Berkeley, CA USA; 2 School of Exercise and Nutritional Sciences, San Diego State University, San Diego, CA USA; 3 Department of Health Promotion, College of Public Health, University of Nebraska Medical Center, Omaha, NE USA

**Keywords:** School health, Physical activity, Implementation science

## Abstract

**Background:**

To address low state physical education (PE) quantity and quality law implementation in elementary schools, the New York City Department of Education (NYCDOE) delivered a multilevel intervention (PE Works; 2015-2019), which included a district-led audit of school PE-law implementation, feedback, and coaching with principals. Using the Reach, Effectiveness, Adoption, Implementation, Maintenance (RE-AIM) implementation science framework, we assessed the primary multilevel drivers of success for this approach in increasing adherence to PE quantity and quality law.

**Methods:**

We conducted in-depth, semi-structured interviews with district-level personnel (*n*=17), elementary school administrators (*n*=18), and PE teachers (*n*=6) in 2020-21.

**Results:**

Interview results suggested several key RE-AIM drivers of successful PE law implementation. Reach: Ensure higher-need schools receive the necessary initial support to improve PE and later focus on lower-need schools. Effectiveness: Provide support tailored to school needs, not penalties, to improve PE. Adoption: Increase the priority of PE at both district and school levels (e.g., audit and feedback, themselves, appear to elevate PE’s priority). Streamline data collection and feedback reports; collecting/reporting too much information is burdensome and leads to lack of focus. Involve qualified (i.e., skilled in both school administration and PE programming/pedagogy) district-level personnel to work collaboratively with schools. Implementation: Build strong, trusting district-school relationships. Maintenance: Provide ongoing district-level support to schools and involve parents to advocate for quality PE.

**Conclusions:**

PE audits, feedback, and coaching (PEAFC) can guide schools in establishing long-term plans for successfully implementing PE-related law. Future research should examine the impact of PEAFC elsewhere (e.g., secondary schools, other districts).

**Supplementary Information:**

The online version contains supplementary material available at 10.1186/s12966-023-01479-1.

## Background

For optimal health, the US Department of Health and Human Services recommends children participate in at least 60 minutes of daily moderate-to-vigorous physical activity (MVPA) and emphasizes the importance of school-based physical activity promotion strategies to achieve this goal [1]. School physical education (PE) has been identified as one of the most valuable, yet underused, opportunities to increase children’s MVPA and improve cardiovascular fitness [2, 3]. PE offers students of all backgrounds opportunities to be active and to obtain the skills and knowledge needed for a lifetime of activity and health-enhancing behaviors [4–8].

As of 2016, 19 US states which were responsible for educating over 13 million elementary students had education laws stipulating the minimum number of PE minutes (*quantity*) students should receive. In addition, 35 states required PE certification/licensure to teach the subject at the elementary level (to help ensure higher class *quality*). Multiple studies have demonstrated students accrue more MVPA and spend more time in standards-based activities when classes are taught by a PE teacher [3, 9, 10]. While the existence of PE laws demonstrates the value of PE, schools often do not comply with such laws due to competing priorities and a dearth of resources (e.g., too few PE teachers) [3, 11, 12].

Elementary schools are less likely to comply with PE state standards than secondary schools, [10] and are thus a key target for interventions that increase adherence to existing PE law. Qualitative evidence from district and school staff suggests interventions to increase PE law compliance are likely to be most impactful when they include an implementation process that engages multiple levels of influence (district, principals, and teachers) and provide in-school support for PE, such as PE teachers, along with teacher trainings and coaching [13–18]. However, little is known about best implementation practices for applying such layered supports to ensure PE is implemented with fidelity to state law [19].

In 2015 only 4% of New York City elementary schools were compliant with the state PE law stipulating students receive at least 120 minutes of PE per week taught by a certified PE teacher [20]. To address low levels of PE law compliance in elementary schools, the New York City Department of Education (NYCDOE), the nation’s largest school district, implemented PE Works [20] – an ambitious, multilevel intervention to improve PE implementation. Facilitated by a large cash infusion, PE Works sought to remove historical system-level barriers to quality PE implementation (i.e. low priority, funding, and expectations for PE) by simultaneously employing several evidence-based interventions [21] at multiple levels. The PE Works intervention included: 1) the provision of state certified PE teachers in elementary schools; 2) increased classroom teacher training in leading PE, [22] and 3) a PE audit and feedback system [23] combined with coaching to help schools with PE implementation and ensure PE teachers had appropriate training.

The PE audit, feedback, and coaching (PEAFC) component of PE Works, which was delivered by district-level staff to participating schools’ principals, is of particular interest as a potentially cost-effective tool for increasing implementation of New York State PE law, as it can be implemented in the absence of the substantial financial resources needed to support hiring of certified PE teachers or in-depth training of classroom teachers.

In school settings, observational evidence suggests PEAFC can successfully support curriculum planning [24] and improve both teaching quality [25] and student academic outcomes [26]. Evidence from California elementary schools suggests audit and feedback could be an effective mechanism for improving PE implementation. For example, audit and feedback in 20 San Francisco elementary schools increased PE by an average of 14 minutes/week (25% relative increase) two years later [23]. Nonetheless, evidence on PE audit and feedback when combined with coaching is lacking. Further, best implementation practices for PEAFC, including targeting of the theoretical processes underlying successful implementation, have yet to be identified.

Using the reach, effectiveness, adoption, implementation, and maintenance (RE-AIM) implementation science framework, [27] we sought to determine the primary drivers of successful PE law implementation through PEAFC. Findings from this study can be used to determine the potential scalability of this approach for improving elementary school PE law compliance in other school districts.

## Methods

### Setting and participants

This qualitative study involved interviews conducted from June 2021-May 2022. Eligibility criteria included being either: 1) an adult educator who worked for the NYCDOE Office for School Wellness Programs (OSWP) as part of the district-level PE Works administrative team during PE Works (school years 2015-16 through 2018-19), or 2) as an elementary (grades Kindergarten through 5^th^) school administrator or PE teacher during PE Works. Study procedures were approved by UC Berkeley’s Committee for the Protection of Human Subjects (#2020-09-13643) and NYCDOE’s Institutional Review Board (#3788). A completed COREQ checklist is provided as supplementary material.

### NYCDOE PEAFC tool

As designed, the PEAFC tool consisted of nine binary (yes/no) PE quantity/quality indicators, including those related to: 1) Presence of a credentialed PE teacher and compliance with state PE requirements; 2) Family-community ties (e.g., communication with families about PE); and 3) Supportive environments (e.g. safe designated PE space). District-level PE employees completed an onsite audit of the nine indicators (through visual assessment and discussion with school administrators/teachers). Once completed, data were entered into a database and a two-page report was produced for each school (see supplementary materials) that: 1) described all nine components assessed; 2) highlighted components in which the school was succeeding; and 3) detailed components that needed improvement with suggestions on how to do so.

District personnel shared this report in a face-to-face meeting with the school principal, providing the opportunity to process the findings, as well as to collaboratively create an action plan for making needed improvements. District personnel then provided ongoing coaching and support for principals to execute their action plan, which ranged from helping create a master PE schedule, to setting up organizational systems for PE equipment. District-level personnel also worked directly with PE teachers providing both group and one-on-one trainings related to topics such as PE curriculum, pedagogical approaches, and behavior management.

### Implementation framework and interview guide

The RE-AIM framework centers on identifying critical intervention components to inform and improve sustainable program adoption and implementation [27]. This framework was specifically chosen because of its systematic approach for identifying both organizational and individual factors as operationalized within the unique, complex, and hierarchical school district environment. To complement this approach, we also used Diffusion of Innovation theory, [28] which refers to the social system process in which people make decisions to adopt a new process or practice. Combing RE-AIM and Diffusion of Innovation theory helped us determine what was driving key personnel at both the district and school levels to adopt and implement PEAFC.

Guided by the RE-AIM framework [27] and Diffusion of Innovation theory, [28] semi-structured interview questions were developed to assess the primary drivers of successful PEAFC implementation separately at the district and school levels. Questions focused on barriers/facilitators to success and impact. Questions assessed 1) historical PE program access in NYCDOE (reach); 2) If/how PEAFC contributed to improved elementary PE and the most effective intervention components for improving PE (effectiveness); 3) Critical components to the successful adoption of PEAFC and what drove adoption decisions (adoption); 4) Key components for successful state PE law implementation through PEAFC, drivers of implementation decisions, and strategies and adaptations for future use (implementation); and 5) Motivation for using PEAFC components over time and programmatic elements continued in schools post-program completion (maintenance).

### Recruitment

Using purposive sampling, we recruited district-level OSWP employees who had worked with PEAFC to participate in interviews via email. School-level participants were recruited first through an email sent by OSWP to elementary school principals who participated in PE Works, inviting them to contact the lead researcher if interested. This approach resulted in only 5 completed interviews (<1% response rate), so the research team next directly called schools to request/schedule interviews. Recruiting stopped once data saturation was reached. Participants provided written consent prior to being interviewed.

### Interviews

Interviews were conducted by Assistant Research Professor and lead author (HRT) who had prior training and experience in conducting qualitative interviews [18, 29–31]. She had worked in collaboration with four of the district-level interviewees to establish the study, but had no relationship with them prior to, or outside of, the research project. She also had no previous relationship with school-level interviewees. Prior to all interviews, she explained the purpose of the study, assured confidentiality, and provided an opportunity to ask any clarifying questions. She also briefly described her prior experience working with schools and districts to improve PE law implementation. District-level interviewees, per NYCDOE IRB regulations, were ineligible for study incentives. School-level interviewees received a $20 gift card for participation. Interviews took place on a single occasion (with no repeat interviews) at the location of the participant’s choice by telephone/Zoom. Because interviews did not take place in person, it is unknown whether anyone other than the participant was present. District-level interviews lasted approximately 20-60 minutes and school-level interviews lasted approximately 15-25 minutes. Brief field notes were taken during the interviews, and with participant permission, interviews were audio-recorded and professionally transcribed verbatim. Transcripts were not returned to participants for comment or correction.

### Data analysis

School-level demographic data were downloaded from the NYCDOE InfoHub (https://infohub.nyced.org/) and analyzed using descriptive statistics in Stata v.16 (StataCorp LLC, College Station, TX). We used a thematic analysis approach [32] to code and analyze interview data using Dedoose Qualitative Software (version 9.0.54, SocioCultural Research Consultants LLC). First, we used deductive coding, with codes based on predefined themes guided by the RE-AIM framework [27] (e.g. reach, effectiveness, adoption, etc) and Diffusion of Innovation theory [28] (e.g. drivers of success). Second, we used inductive coding to accommodate sub-themes that emerged from the data (e.g. specific barriers/facilitators to success and areas of impact). Each transcript was independently coded by two researchers (HRT and MZ) using an iterative process, with the researchers refining and consolidating codes and adding sub-codes and emergent codes as needed. Once the coding structure stabilized and saturation was reached (defined as no new codes were found in the data), [33] the researchers applied final codes to all transcripts. Once transcripts were double-coded, the coders met to review all transcripts to ensure consistent application of codes throughout. If disagreements occurred, the coders revised and refined until agreement was reached [34]. Lastly, the researchers came to a consensus on final themes/subthemes and identified drivers of successful implementation across both district- and school-level interviewees. Participants did not provide feedback on the findings.

## Results

A total of 41 adults were interviewed (Table 1). District-level interviewees held Director or Associate Director positions (41%), followed by Executive/Senior Directors (35%), and Manager/Specialists (24%). On average, they had 14 years of experience in the NYCDOE and nine years in their current position. Three-fourths (75%) of school-level employees interviewed were school administrators (Principals or Instructional Coaches) and 25% were PE teachers. School-level employees had an average of 23 years working in the NYCDOE and 10 years in their current position. They worked at 21 different elementary schools (both an administrator and a PE teacher were interviewed from three schools).

**Table 1:** Characteristics of New York City Department of Education (NYCDOE) district- and school-level personnel interviewed (*n*=41)

	**District-level (** ***n*** **=17)**	**School-level (** ***n*** **=24)**
Male, n (%)	3 (17.7)	9 (37.5)
Female, n (%)	14 (82.4)	15 (62.5)
Primary position during PE Works (2015-16 to 2018-19), n (%)		
Executive/Senior Director	6 (35.3)	-
Director/Associate Director	7 (41.2)	-
Manager/Specialist	4 (23.5)	-
School Administrator	-	18 (75.0)
School PE teacher	-	6 (25.0)
Years in NYCDOE, mean ± SD		
Executive/Senior Director	14.8 ± 5.4	-
Director/Associate Director	10.0 ± 4.8	-
Manager/Specialist	17.5 ± 5.8	-
School Administrator	-	23.0 ± 6.5
School PE teacher	-	19.7 ± 7.6
ALL	13.5 ± 5.8	22.5 ± 6.6
Years in position, mean ± SD		
Executive/Senior Director	7.8 ± 3.6	-
Director/Associate Director	7.3 ± 1.3	-
Manager/Specialist	12.5 ± 7.8	-
School Administrator	-	10.7 ± 4.7
School PE teacher	-	7.0 ± 4.2
ALL	8.5 ± 3.9	9.9 ± 4.8

Average enrollment in the 21 schools was 518 students (98.9% non-white) with 76% qualifying for free or reduced-price meals (a proxy for household socioeconomic status). There were no significant school-level demographic differences between schools whose administrators/PE teachers were and were not interviewed for this study (Table 3)

Overall, PEAFC was considered a success by the district, with interviewees unanimously agreeing the program significantly improved PE implementation and adherence to state elementary PE law. PEAFC did so by providing a structured means for NYCDOE district-level personnel to work one-on-one with schools to identify the current strengths and weaknesses of their PE program. PEAFC also enabled district personnel to provide direct feedback and specific supports to schools that were necessary to help ensure schools implemented programs in adherence with state quantity and quality law.

Using the Reach, Effectiveness, Adoption, Implementation, Maintenance (RE-AIM) evaluation framework, [27] we present the drivers of success and subsequent themes for the district and school level key stakeholders. Themes and sub-themes are presented in Table 2. Illustrative quotes are presented in Appendix Table 1.

**Table 2:** Description of reach, effectiveness, adoption, implementation, and maintenance drivers of success, facilitators and barriers, and impact across district- and school-level personnel who administered and/or worked with the PE Works Audit and Feedback (PEAFC) tool in the New York City Department of Education (NYCDOE)

**District-level administrators (** ***n*** **=17);** Facilitators (+), barriers (–), impact (•)	**School-level administrators/PE teachers (** ***n*** **=24);** Facilitators (+), barriers (–), impact (•)
**REACH, Driver of Success:** First ensure highest need schools receive necessary attention/support, then move on to lower-needs schools	
+ Prioritized highest need schools during pilot cohort, which included more focused time and attention on highest needs schools – Students not reached because PE was not a priority at that school, relative to other subjects – Students not reached because principal had prior bad personal experience with PE • PE Works audit and feedback reached students across schools, districts, and boroughs, regardless of school-level demographic characteristics prior identified as associated with poor PE quantity	+ Principal is open and willing to accept the help to improve PE + School community, including teachers and parents, is bought-in – Students not reached because PE was not a priority at that school, relative to other subjects • PE audit and feedback increased reach of PE program within the school • Improved PE impacted the entire school community
**EFFECTIVENESS, Driver of Success 1:** Ensure everyone is on the same page: a unified goal is necessary for PEAFC effectiveness.	
+ Using audit and feedback as a tool to communicate about, and advocate for, PE + To meet goal, need to first assess and identify needs and next support schools in meeting needs • Audit and feedback helped increase administrators understanding of PE programming and its importance	+ To meet goal, need to embed PE structures and processes in school • Audit and feedback helped increase administrators understanding of PE programming and its importance
**EFFECTIVENESS, Driver of Success 2:** Meet schools where they are and provide tailored on-the-ground partnership and supports for improving PE	
+ Supported, rather than penalized, for being non-compliant with PE law + Laid out clear components for successful PE program + Supports were structured, layered, and gradual (change takes time) • Helped schools develop a personalized plan for establishing components of strong PE program	+ Supported, rather than penalized, for being non-compliant with PE law + Laid out clear components for successful PE program – Without plan and supports, audit and feedback wouldn’t be as effective • Helped schools develop a personalized plan for establishing components of strong PE program
**EFFECTIVENESS, Driver of Success 3:** Provide schools with a PE teacher(s) and the appropriate teacher(s) supports, for PEAFC to be as effective as possible.	
+ Schools need help in identifying qualified teachers and then in supporting them + Initial full funding of PE teacher was critical to get foot in the door + Create or ensure a strong, navigable pipeline from PE credentialing programs to schools, so that there is a pool of eligible teachers for schools to choose from • Helped schools who wanted and needed a PE teacher find someone who fit well within that school and provided adequate and appropriate training and support for new PE teachers	+ Helping schools identify qualified teachers and then provide them resources + Providing trainings and continued professional development for PE teachers + Professional learning communities help support PE teachers establish effective programs – Tell schools to get a PE teacher but don’t help them in finding or training that teacher • Helped schools who wanted and needed a PE teacher find someone who fit well within that school • Provided adequate and appropriate trainings and support for new PE teachers
**ADOPTION, Driver of Success 1:** PE needs to be a priority at the district level, which in turn drives priority at the school level	
+ Audit and feedback needs to be prioritized and funded at the district level + If PE is valued by district, it will increase priority in schools, making adoption more likely + PE needs to be prioritized/valued across the school (not just by principal/PE teacher) + There needs to be a clear vision for what PE should look like both across the district and within schools • Program demonstrated increased district-level PE priority that was felt at school level • Priority for PE was increased in most PE Works schools	+ Seeing PE prioritized by superintendents and district-level personnel increases importance of PE within schools. + Still, many principals need support in making PE a priority at their school + Receiving funding for PE (via PE teacher salary, equipment, professional developments, etc.) increases priority for PE in schools + Initial funding for PE teacher critical to getting program started in schools • Program demonstrated increased priority for PE at the district level that was felt at the school level and priority for PE was increased in most PE Works schools
**ADOPTION, Driver of Success 2:** Need appropriate, actionable indicators for auditing PE programs	
+ Data should be collected as part of a conversation with principals, not just reading off survey questions + Positive data (things school is doing well) should be highlighted as much as what needs to be worked on. + Collect data for schools themselves, but also to indicate need across districts – Too much data can lead to paralysis. (i.e. collecting data on nine components is too much); limit to only key components if personnel or resources are limited • Actionable indicators helped schools clearly see their PE-related strengths and deficiencies, and forms strong foundation for technical assistance work to help address the deficiencies	+ Collect data and report on PE indicators that can be improved + Don’t just identify the problem, help schools solve it – Indicating lack of space is frustrating because it’s not easily actionable for NYC schools • Actionable indicators helped schools clearly see their PE-related strengths and deficiencies, and forms strong foundation for technical assistance work to help address the deficiencies
**ADOPTION, Driver of Success 3**: Need an efficient data entry system and automated process for producing feedback reports	
+ Need to train district personnel on quality data collection and data entry + Data entry needs to be user-friendly for non-techy district-personnel + Manual work to ensure feedback/action plans are appropriate for each school + Strong template helps, but personalization is often needed – Limited capacity at the district-level to analyze data across district Streamlined data entry and reporting system enabled district to provide structured feedback to schools • Too much data and not enough people at the district level to help analyze the data led to some inaction.	N/A
**ADOPTION, Driver of Success 4:** Need appropriately qualified and passionate district-level personnel who are working collaboratively to work with schools	
+ Need district personnel who know school administration + Need district personnel who know PE pedagogy and teaching + Need district personnel who can work with data + Having a smaller caseload of schools, so district personnel can spend more time with each school and really get to know the school and its community + Need coordination of/trainings for district-level teams so everyone is on the same page • Teams of district personnel (one with an administrative background, one with a PE background) worked well together to support both principals and PE teachers through the audit and feedback process	+ Need district personnel who know school administration, so they understand challenges of principal in overseeing the execution of a strong PE program + District personnel having prior in-school experience is very helpful + Need district personnel who know PE pedagogy to directly help the PE teacher • Teams of district personnel (one with an administrative background, one with a PE background) worked well together to support both principals and PE teachers through the audit and feedback process
**IMPLEMENTATION, Driver of Success 1:** Need a personalized assessment of, and structured plan for, each school, which includes proactive coaching to improve PE	
+ Use real, relevant data because principals value data on their school + Plan to improve PE and supports need to be targeted to each individual school + Plan needs to meet schools where they are and have realistic expectations + Plan needs to be layered to evolve over time + Coaching is proactive, as opposed to reactive + School-level supports are available, personalized, and helpful – Changing PE is a journey that takes time; rushing shows you don’t understand schools • Schools received a structured, personalized plan to improve PE, which, when followed and supported, enabled them to significantly improve their PE programs • Technical assistance and layered related supports led to improved PE quantity/quality	+ Use real, relevant data because principals value data on their school + Plan to improve PE and supports need to be targeted to each individual school + Plan needs to meet schools where they are and have realistic expectations + Plan helps schools check progress and see gains + Coaching is proactive, as opposed to reactive + School-level supports are available, personalized, and helpful – Changing PE is a journey that takes time; rushing shows you don’t understand schools • Schools received a structured, personalized plan to improve PE, which, when followed and supported, enabled them to significantly improve their PE programs • Technical assistance and layered related supports led to improved PE quantity/quality
**IMPLEMENTATION, Driver of Success 2:** Building strong district-school relationships is necessary for successful PEAFC implementation	
+ Understanding schools’ unique complexities helps build trust + Need to build trust with the principal + Persistence and patience are key in building relationship + Need to understand complex nature of schools – Being judgmental or hounding a school repeatedly/being smothering • District personnel built strong and lasting relationships with school administrators and PE teachers that helped ensure successful implementation • Coaching and supports led to improved PE quantity/quality	+ Need to build trust + District-level personnel needs to be available + Need balance between being available and not smothering + Follow-up with principal is key + Need to be flexible – Hounding a school repeatedly/ being smothering • District personnel built strong and lasting relationships with school administrators and PE teachers that helped ensure successful implementation
**MAINTENANCE, Driver of Success 1:** District-level personnel should remain available to schools to continue to support PE past the life of the program	
+ District personnel maintained relationships with schools and provided support throughout program and past life of program – Funding was available only for one audit per school • District personnel continued to support schools past the life of the program, which helped schools to maintain strong PE programs	+ Intensive support was good at program start, not necessary once program was established + District personnel were available, if needed, to help support schools once program was complete • District personnel continued to support schools past the life of the program, which helped schools to maintain strong PE programs
**MAINTENANCE, Driver of Success 2:** PEAFC should provide a long-term plan and structure for schools to successfully build and maintain a PE program	
+ Improved PE, and its impact on the students and school community, motivated maintenance of a strong PE program + PE teachers took on leadership roles within schools, which motivated maintenance • Most schools developed and maintained a long-term vision for PE and the structure to facilitate the maintenance of a strong program, which lasted beyond the funded length of the intervention	+ Improved PE, and its impact on school community, motivated maintenance + Student buy-in, including increased expectations for PE, motivated maintenance + Parent and family buy-in motivated maintenance + Integrating PE across departments helped with maintenance • Most schools developed and maintained a long-term vision for PE and the structure to facilitate the maintenance of a strong program, which lasted beyond the funded length of the intervention

### Reach

Reach assessed whether PEAFC reached the target population (NYCDOE elementary school students). While we did not assess reach using the number and representativeness of the students reached, in the traditional quantitative RE-AIM sense, [35] we evaluated reach by analyzing the overall goal of PEAFC and whether or not students in schools, regardless of background, were impacted.

#### Reach driver of success: First ensure highest need schools receive the necessary attention and support, and then move on to lower-needs schools

In planning and implementing the program, district-level personnel intentionally started the first year with a cohort of 50 schools that were considered high-need (based on prior poor PE performance, as well as available student public health-related data) and required the most support for improving PE implementation. This approach helped ensure students across schools, regardless of characteristics historically associated with poor PE law quantity/quality adherence, were universally reached. While wide reach was noted, district and school administrators alike mentioned the tremendous pressures schools are under and the high number of competing priorities, which did impact reach in a handful of schools. Despite the PE Works programmatic supports being offered, according to both district personnel and school principals, the primary reason students weren’t reached was because PE was still not a priority, relative to other subjects (academic and the arts), at some schools.

### Effectiveness

Effectiveness identified how PEAFC contributed to improved elementary PE in the NYCDOE and identified the most effective components of the tool for achieving its goal of improved elementary PE.

#### Effectiveness driver of success 1: Ensure everyone is on the same page: a unified goal is necessary for PEAFC effectiveness

District-level personnel interviewed were able to clearly articulate the goal of PEAFC (i.e., improve elementary PE by first identifying schools’ needs and then providing a plan and supports so schools could meet those needs). At the school level, this was operationalized by district personnel working with principals and PE teachers to embed PE structures and processes into the school (e.g. creating master PE schedules which allowed small physical spaces for PE to be shared effectively; developing PE equipment storage and sharing systems, etc.). At the district level, personnel also used PEAFC as a way to communicate/advocate for the importance of PE. School-level personnel corroborated that PEAFC helped increase administrators’ understanding of the importance and value of PE, as well as the logistics of running a strong program.

#### Effectiveness driver of success 2: Meet schools where they are and provide tailored on-the-ground partnership and supports for improving PE

At both levels, a primary driver of effectiveness was supporting (rather than penalizing) schools that were non-compliant with PE law. At both levels, clearly laying out the components of a successful PE program and helping schools develop personalized plans for establishing those components were critical to effectiveness. District personnel noted these were best accomplished by including supports that were structured, layered, and gradual, as real change within the school environment takes time. At the school level, administrators said that simply auditing schools, without providing feedback in the form of an action plan and supports for that plan, would not have been nearly as effective.

#### Effectiveness driver of success 3: Provide schools with a PE teacher(s) and the appropriate teacher(s) supports, for PEAFC to be as effective as possible

District and school personnel noted that the single most effective component of PE Works was the provision of a certified PE teacher in schools. With this model, the OSWP helped schools recruit and hire needed teacher(s). The district started with a tiered funding structure, in which the district paid for 100% of the teacher the first year, 75% the second, 50% the third, and 0% the fourth, with the school resuming responsibility for non-district funding. According to principals, this funding structure was highly effective. Further, not just providing PE teachers, but ensuring they had the supports they needed was also critical. The district facilitated Professional Learning Communities for PE teachers, to facilitate collaboration and group learning, which was universally appreciated by PE teachers who often feel siloed within their schools as the only PE teacher for whom many traditional in-school professional learning opportunities do not apply. While several interviewees noted that PEAFC, absent the provision of a PE teacher, could be effective for improving PE, it was hard to disentangle the valuable role of the PE teacher. Insufficient funding for PE teachers in other school districts could be a barrier to successful PEAFC implementation elsewhere.

### Adoption

Adoption determined which specific PEAFC components interviewees believed were critical to the successful adoption of PEAFC in elementary schools. We also examined what drove the adoption decisions being made at both the district and school levels.

#### Adoption driver of success 1: PE needs to be a priority at the district level, which in turn drives priority at the school level

The primary factor driving PEAFC adoption was the prioritization of PE at the highest systemic levels (first the city, with the Mayor’s office, then City Council, then the NYCDOE), which resulted in direct funding for the program. Priority setting in the NYCDOE system starts with the mayor, and then filters down through the NYCDOE Chancellor, and so on. Both district- and school-level personnel agreed because PE was a priority at the district level, it became a greater priority within schools. Further, because PE Works came with direct funding, it drove adoption. The district-level (i.e. funding for OSWP personnel to support schools) and school-level funding (i.e. PE teacher salaries, professional development) directly helped principals set PE as a priority in their schools.

#### Adoption driver of success 2: Need appropriate, actionable indictors for auditing PE programs

District-level personnel described collecting real, actionable data from schools related to PE as another primary driver of adoption. At the school level, principals felt strongly that collecting data on factors that could be improved, versus focusing on things that were not easily changed (e.g., limited space for PE, which is challenging and very costly), helped drive adoption. Similarly, not just identifying problems, but helping schools solve them, drove adoption. District personnel also described being able to aggregate the data across districts or boroughs being very helpful to describe the state of PE and to highlight broader needs and determine if programmatic goals were being met.

Several district level personnel mentioned the PE audit had too many questions on it. For successful adoption in another school district, they recommended cutting back on indicators to facilitate better back-end data analysis and action planning. Specifically, district leaders recommended narrowing down from nine indicators to five and focusing on: school having a PE teacher; having adequate PE minutes scheduled; having PE observed to meet state standards; having PE teacher participate in professional development; and community building around PE and wellness.

#### Adoption driver of success 3: Need an efficient data entry system and automated process for producing feedback reports

Successful PEAFC adoption was also driven by efficient data management and processing systems. Training district personnel on how to systematically collect and enter data was necessary. This has challenges, as it was often noted that being good at interfacing with and understanding schools and being good with data/data entry are not always complementary skills. Creating a template for the feedback reports helped the district present unified messaging across schools, but backend work was often necessary to ensure the feedback reports and action plans were appropriately personalized for each school. Several district-level personnel noted having increased capacity at the district-level to better analyze data would be useful.

#### Adoption driver of success 4: Need appropriately qualified (with both school administration and PE programming/pedagogy skills) and passionate district-level personnel who work collaboratively at the district-level to work with schools

Working with school administrators and PE teachers to improve PE requires two, often distinct, skill sets: school leadership/administrative skills and PE programming/instructional skills. As such, audits were conducted by teams of two district personnel at each school (one with a school administrative background, one with a PE instructional background). It was particularly effective when district-personnel had prior administrative experience in schools, as it helped them both better relate to the principal, but also demonstrated they truly understood the unique challenges of working in the school environment. With multiple auditors working at the district level, training was necessary to ensure personnel were systematically collecting reliable data. Having a smaller caseload of schools, so that district personnel could spend more time working with schools, was also identified as a driver of successful adoption.

### Implementation

Implementation describes district- and school-level administrators use of key components of PEAFC, including implementation strategies and adaptations necessary for the success of PEAFC in future educational settings. We also examined what drove the implementation decisions being made regarding PEAFC at both the district and school levels.

#### Implementation driver of success 1: Need a personalized assessment of, and structured plan for, each school, which includes coaching to improve PE

Interviews demonstrated the importance of data for principals. Because PEAFC utilized personalized school-level data, as well as individualized action plans directly based on that data, implementation was successful in most schools. Having action plans that met schools where they were, that were realistic and appropriately-paced, that evolved over time, and that helped schools check and see their progress, all helped drive successful implementation.

Another critical driver of PEAFC implementation was the coaching, or school-level supports, that were layered in place as part of the action plan. Across both levels, interviewees noted that the appropriate school-level supports need to be available, personalized, and helpful for successful implementation. In particular, coaching was proactive (available and offered to all principals and PE teachers), rather than reactive (in which participants would have had to ask for the help themselves). Another important layer of district-provided support was the NYCDOE’s Move-to-Improve [22] program, which is an evidence-based training and curriculum for classroom teachers to supplement PE under certain conditions, which made it possible to provide daily PE minutes if schools didn’t have enough PE teachers.

#### Implementation driver of success 2: Building strong district-school relationships is necessary for successful PEAFC implementation

One of the most critical drivers of successful PEAFC implementation was a strong and trusting relationship between district- and school-level personnel. District personnel needed to be flexible, patient, persistent, and non-judgmental when building a relationship with the principal. In particular, district personnel’s understanding of the unique and complex issues within public schools, drove implementation. School principals noted that district personnel needed to be available, but not smothering or hounding; they needed to help principals prioritize and operationalize PE, but also understand that principals were simultaneously dealing with multiple competing priorities.

### Maintenance

Maintenance determined motivation for using PEAFC over time and identified programmatic elements school administrators and teachers continued in their schools once the program as technically completed.

#### Maintenance driver of success 1: District-level personnel should remain available to schools to continue to support PE past the life of the program

While the PE audit and feedback process was only implemented once due to funding, many schools maintained a strong relationship with their district partner for PE; district personnel continued to provide coaching and support for PE both over the course of the four-year program, and beyond. By design, the most intensive programmatic supports were available during the first few years of the program, to help get PE up and running, and then waned once schools had an established program. These relationships were maintained because district-level personnel continued to provide valuable services and supports for schools.

#### Maintenance driver of success 2: PEAFC should provide a long-term plan and structure for schools to successfully build and maintain a PE program

Improved PE, and its impact on the school community, clearly motivated maintenance. Specifically, PE teachers taking on leadership roles in the schools motivated principals to maintain PE programs because they provided a clear benefit. Once strong PE programs were established, students and families started to expect a strong program, which further motivated maintenance. When parents became bought into PE, they became PE advocates, which motivated principals to maintain strong programs.

## Discussion

This study used an implementation science framework (RE-AIM) and Diffusion of Innovations theory to identify the drivers of successful implementation of state PE quantity and quality law through development and provision of a PEAFC program. Importantly, PEAFC was well received and appreciated by NYCDOE district- and school-level personnel alike. PEAFC provided a structured means for district-level personnel to work directly with schools to identify the current strengths and weaknesses of the PE program, and to then provide the direct feedback and specific supports necessary to help ensure implementation of PE programs in adherence with state quantity and quality law. A similar approach has been used successfully in several other health intervention settings, in which coalitions go through a continuous process of monitoring, data-sharing, planning, and action with technical support [36–38] However, ours is the first known study to evaluate this approach related to PE law implementation in the school district context. Lessons learned from NYCDOE can be used to guide adaptation of PEAFC in other districts.

These findings suggest PEAFC can be an effective means for improving PE law implementation, a subject with a long history of being under-prioritized in the elementary school setting [39, 40]. In NYCDOE, PE Works addressed a critical and identified gap across city elementary schools; at both the district and school levels, interviewees recognized and appreciated the importance of providing quality PE. However, prior to the program, they had lacked the systems and resources to ensure successful implementation. Evidence from other states similarly demonstrates that schools require substantial supports in adhering to PE laws; passing a law, alone, does not mean it will be implemented [41, 42]. However, prior school-based PE research has focused primarily on approaches to either increase quality (e.g. increasing student MVPA through curricular changes or classroom teacher training to teach PE [9, 43, 44]) or on quantity (e.g. adherence to PE quantity law through a litigious approach [29, 45]), alone. No other known PE-specific intervention has been identified to increase adherence with state PE quantity and quality law simultaneously.

One strength of PE Works’ approach was the district funding to help initially support schools in hiring a certified PE teacher. Prior research confirms that PE teachers are among the top things principals want and need to improve both PE quantity and quality [3, 29, 40]. While both district- and school-level personnel reported that PEAFC alone is less likely to be effective without a certified PE teacher and supports for that teacher, many believed that PEAFC on its own does have potential to positively impact PE. This corroborates prior evidence from California elementary schools, wherein there was a 25% relative increase in weekly PE minutes two years after publicly disclosing PE law compliance data collected in San Francisco schools, [23] and a 53% relative increase in weekly PE minutes after PE-related data were collected in Sonoma County schools and shared with education leaders [46]. Both San Francisco and Sonoma schools saw these changes absent substantial district-wide funding for additional PE teachers. Without funding for PE teachers, PEAFC has the potential to be successful in other school districts, but more research is necessary.

Using an approach that ensures equitable PE programming is provided across schools is an important driver of reach. In NYCDOE, this involved targeting the highest-needs schools to participate in the first cohort of PE Works schools, to ensure PEAFC could be successful in historically more challenged PE settings. In NYCDOE, as well as nationally, prior evidence demonstrates that schools with a higher proportion of students from historically marginalized communities are least likely to provide strong PE programs, to have PE teachers, and to have students who meet cardiorespiratory fitness standards [3, 11, 47]. Targeting under-performing schools is a common approach in academic interventions aimed at closing the achievement gap [48]. Using local data to target schools which have long been under-served and under-resourced to provide high quality PE is an important way to ensure equitable PE reach.

Building strong district-school relationships is an important driver of successful PEAFC effectiveness, adoption, and implementation. For success in future districts, 1) having appropriately qualified and passionate district-level personnel working collaboratively with schools; 2) ensuring principals are willing to make time for PE, hear feedback, and take action; 3) having a personalized assessment of, and structured plan for, each school; and 4) providing tailored supports, including coaching, for improving PE should help drive successful implementation. As with many school-based, real-world interventions, [49] a one-size-fits-all model clearly would not have worked in NYCDOE to improve PE, which is likely the case in most school districts. Instead, having a deep toolkit of available supports for on-the-ground PE implementation, which can be flexible depending on each school’s unique needs, will likely help drive implementation and programmatic success in future settings.

In particular, the coaching component of NYCDOE’s approach was critical to success; audit and feedback, alone, would likely not have yielded such positive results. Prior evidence suggests a proactive, rather than responsive, approach to coaching is ideal, as coaching recipients with fewer skills are less likely to make requests for help [50, 51]. PE Works’ proactive coaching approach, in which district personnel were both responsive to requests for assistance, as well as anticipatory, helped facilitate the implementation process.

Having district personnel available to support schools past the life of PEAFC facilitates program maintenance. Depending on personnel time dedicated to PE at the district-level, this could be a challenge elsewhere. However, what appeared to be an even greater driver of maintenance was that PEAFC provided a long-term vision and structure for schools to successfully build and maintain a PE program. It was clear that PE Works wasn’t seen as a “Band-Aid” solution to poor PE law compliance, wherein a program comes in and is successful when funding is present but conditions revert to pre-program once funding has ended. On the contrary, many schools reported needing that initial, concentrated support at the beginning of the program. But then, being able to run PE on their own several years in, because they had taken the time and received the supports to build a strong program. Parental support for quality PE, once established and recognized as important, additionally became an important driver of program maintenance; once families began expecting high-quality PE, they pressed on principals to ensure programming continued.

Study limitations deserve mention. First, interviews took place 6-7 years after PE Works started and 2-3 years after the program ended, which could contribute to recall bias. Delays in the research were primarily related to the COVID-19 pandemic, which limited access to conducting research in NYCDOE. In addition, the population interviewed may not fully represent the entire population of elementary administrators in the NYCDOE or elsewhere. However, Table 3 data suggest interviewed principals were from schools demographically representative of the city’s elementary schools.

**Table 3:** 2020-21 School year demographic characteristics of New York City Department of Education (NYCDOE) elementary schools whose administrators/PE teachers were and were not interviewed for this study (*n*=695 schools)

	Interviewed (*n*=21 schools)	Not interviewed (*n*=674 schools)	*p*-value for difference^A^
Total enrollment, mean ± SD	518 ± 237	504 ± 252	0.7966
Female students, % ± SD	49.7 ± 2.7	49.2 ± 3.3	0.4420
Asian American students, % ± SD	17.8 ± 22.1	15.0 ± 19.8	0.4671
African American students, % ± SD	24.2 ± 27.1	25.8 ± 26.0	0.7782
Hispanic/Latinx students, % ± SD	38.0 ± 23.0	41.8 ± 26.0	0.5071
White students, % ± SD	1.1 ± 1.3	1.3 ± 2.7	0.8215
Multi-racial students, % ± SD	1.9 ± 3.2	1.8 ± 2.5	0.8663
Students with individualized education plans, % ± SD	19.7 ± 4.4	20.5 ± 7.6	0.6519
Students who are English-language learners, % ± SD	14.2 ± 12.0	15.1 ± 11.5	0.7173
Students who qualify for free or reduced-price meals, % ± SD	70.5 ± 25.7	74.2 ± 22.7	0.4595

^A^ *P*-values for difference between interviewed and not interviewed schools derived from unpaired t-tests

## Conclusion

This study provides first-of-its-kind evidence on, and drivers of, successful PE law implementation through engaging a multilevel system of district- and school-level administrators and educators on critical PEAFC reach, effectiveness, adoption, implementation, and maintenance processes. This study outlines promising practices for scaling PEAFC to additional school districts. Future research examining the impact of PEAFC outside of NYCDOE, with a particular focus on examining the impact of PEAFC absent other PE-related interventions (i.e., funding for PE teachers) is an important next step in identifying tools for increasing PE law implementation and improving related youth health outcomes.

## Supplementary Information

**Additional file 1: Appendix Table 1. **Illustrative quotes associated with description of reach, effectiveness, adoption, implementation, and maintenance (RE-AIM) themes across district- and school-level personnel who administered and/or worked with the PE Works Audit and Feedback (PEAFC) tool in the New York City Department of Education (NYCDOE)

**Additional file 2.**

## Data Availability

The qualitative datasets generated and analyzed during the current study are not publicly available, as they could compromise individual participants’ identities, per the research agreement with the New York City Department of Education Institutional Review Board. However, select qualitative data are available from the corresponding author on reasonable request. School-level demographic data used in Table 1 of this study are publicly available at https://infohub.nyced.org/reports/school-quality/information-and-data-overview.

## Abbreviations

PE: Physical Education
NYDOE: New York City Department of Education
PEAFC: PE audit, feedback, and coaching
MVPA: Moderate-to-vigorous physical activity
RE-AIM: Reach, effectiveness, adoption, implementation, and maintenance, implementation science framework
OSWP: NYCDOE Office for School Wellness Programs
