# Supplementary material for: Implementation of elementary school physical education quantity and quality law through school district audit, feedback, and coaching
Source: Int J Behav Nutr Phys Act. 2023 Jun 29;20:77. doi: 10.1186/s12966-023-01479-1 (PMC10308623; doi:10.1186/s12966-023-01479-1)
Supplement: Supplementary file 1 — Additional file 1: Appendix Table 1. Illustrative quotes associated with description of reach, effectiveness, adoption, implementation, and maintenance (RE-AIM) themes across district- and school-level personnel who administered and/or worked with the PE Works Audit and Feedback (PEAFC) tool in the New York City Department of Education (NYCDOE) [file 12966_2023_1479_MOESM1_ESM.docx]

Appendix Table 1: **Illustrative quotes associated with description of reach, effectiveness, adoption, implementation, and maintenance (RE-AIM) themes across district- and school-level personnel who administered and/or worked with the PE Works Audit and Feedback (PEAFC) tool in the New York City Department of Education (NYCDOE)**

| **District-level administrators (n=17)** | **School-level administrators and PE teachers (n=24)** |
| --- | --- |
| **REACH, Driver of Success:** First ensure highest need schools receive necessary attention/support, then move on to lower-needs schools | |
| - So we used the equity data to identify universal schools that were high need that were not meeting PE requirements that we may or may not have funded in some way in the past. And we invited these schools to partner with us. And by partnering with us, the vision was that all the programs and all the instructional and programmatic support that we offer will be targeted for these partnered schools to see what the impact would be if we work with the school because, in the past, our programs have been separate like come apply for X funding and it was never sort of crossing across the neediest group of schools. | - But [PE] it's really more about the whole child, the whole school, and the whole community. So, I think once you have buy-in for what it can be, then it makes it so much easier to actually implement because once you have that admin support, they know the why, they know the impact, and the ultimate outcomes that they can have, it's just easier to move to move it along. |
| **EFFECTIVENESS, Driver of Success 1:** Ensure everyone is on the same page: a unified goal is necessary for PEAFC effectiveness. | |
| - “The goal was to collect data on what schools actually had in place and gaps, in other words, challenges that they were facing and in terms of staffing, programming, space and then a community, family engagement, etc. And to take that data and then tailor an approach for each school, tailor a way to work with them so that each school would be getting support that was specific to their needs.” - “So I think the intent was for us to have a true analysis of what was happening at the school, to be able to then, you know, have an understanding of some of the structures that are needed to move PE instruction, whether it’s environmental, instructional, structural, whatever it could be, to then help that school in a more targeted structure on how we would support them to make those changes systemically within the school.” | - “It was helpful in helping me, as an admin, learn a bit more about the expectations and standards around PE. So that when I was meeting with my PE teacher or doing an evaluation of a PE class and offering feedback, or when I was trying to understand the state’s expectations or the instructional expectations of the [district], it helped me to go through that [audit and feedback] process to better understand the PE program.” - We have embraced it and made it a cornerstone now of what we do. And until you actually apply it and do it correctly, I didn’t see the value in PE. It may not seem like you’re getting a lot from it at first, but when you see how the kids’ attitudes change about [PE], and the pride the kids have in the school, it is something. So, what I find is it keeps the entire community happy because the kids are engaged, the parents see that their kids are happy, so I just feel like it gets me a lot of buy-in and capital with my community.” |
| **EFFECTIVENESS, Driver of Success 2:** Meet schools where they are and provide tailored on-the-ground partnership and supports for improving PE | |
| - “It was successful because we said, how do we identify the different components that go into making a really quality PE program for kids? And then how can we help your teacher, the administrator, start to work on those? So, I think it was that, how do we build this together? We’re not just saying, like, this is all the stuff you need to do, or you’re not hitting 120 minutes, everything is horrible. I’m saying, fine, okay, so you’re not in 120 minutes, but we can help you get there.” - “So, it helped us be much more consistent and organized, I think efficient in working with schools. I think it gave us a good communication tool that administrators/teachers—we were able to develop and start to really speak the same language. I think it made the work seem a bit more doable. There are multiple points of entry and ways I think for us to get some early wins and for the schools to feel like they are getting some early wins because we have this tool. And it helps to make sure that they’re moving along. If the school’s good in some certain aspects, great. We want to help them maintain that. But more of our energy should be focused on the areas where it’s more of a challenge, that they need more support, or we’re not going to see as much movement.” | - “When you bring PE Works into your school, what you’re doing is bringing in a critical partner, and a partner that is not going to be more work for you, but who will actually help you to be able to do other things – without having to worry about compliance with PE law and worrying about programming with PE…And the reality is that there’s no getting around it. We are required by state laws to provide physical activity to the children. So, if we’re required to do it, then you might as well have people in place that can support you to do this work.” - “I found both of it useful, having the initial conversation about where my school was because most principals are not willfully not complying with expectations for PE. You aren’t just saying, oh, well, I’m not going to do that. You’re just working with the reality of who and what you have. And so sometimes the noncompliance is just a matter of default, and not because it’s a deliberate attempt on our part not to meet the expectations. But just having the conversation, and even troubleshooting to say, well, if these are all the negatives, can you offer suggestions for this until we can get the compliance fully in place, right. This scaffolding and sort of like the gradual release of, okay, so here’s a small targe for you to work on, and then you can do this, and then you can do that.” |
| **EFFECTIVENESS, Driver of Success 3:** Provide schools with a PE teacher(s) and the appropriate supports for that teacher(s), for PEAFC to be as effective as possible. | |
| - “So if the school did not have a certified teacher, then maybe identifying one teacher who might have had a background in education and sport education and say, Would you be willing to at least go to professional development until the school can get the funding to get a certified PE teacher.” - “I know that the money piece and the hiring of teachers is a difficult component for other districts. But with that, the principals saw the value. So, when we placed these licensed PE teachers who we worked to develop, they saw the difference. They saw the value in having that individual in their school and that led to retention, which led to schools wanting to keep them and wanting to hire more PE teachers.” | - One of the frustrations of being a principal is that even if you have the PE vacancy, is finding a certified qualified person. And they help you do all that, so that’s a big plus, plus the professional support they provide, is very important because you can get a good candidate, but they might need help in being effective in their work. And then that shows the value to the principal of having this person. - Where I was able to hire an individual who was passionate and more of an expert,I think that made a big difference for us and I don’t know if it would have made the difference without that teacher. I just feel like the key lies in the people. And if the person is passionate and knowledgeable, the investment is going to go a lot further.” |
| **ADOPTION, Driver of Success 1:** PE needs to be a priority at the district level, which in turn drives priority at the school level | |
| - So generally speaking, I feel like there was a shift in central leadership of the mentality of wellness. So I feel like that was very successful. And then I did a lot of work with supervisors during this time, when it first kind of rolled out, which I think was critical. We sat in an office with one of the superintendents of the districts we supported, and she was fully on board, fully prepared to advocate for this work and speak to principals on behalf of it to show what the need was. So that was super helpful you could see the difference in the districts where that existed.” - “However, it's from the top-down. Whoever's making decisions with funding and whoever starts the road map, if they see physical education as a priority, then that will trickle down because all the policies, the directors, the funding will be geared towards that sort of priority.” - I think you need a really clear vision of where you're going, not just for the first initial initiative of three years or whatever. But to really, even if it's just roughly mapping it out so that after the first year, after the second year, you can come back and say, what did we think, how did we do?” | - “Before PE Works, you could so easily tell PE wasn’t a priority. But it is now.” - “If we look at what PE looked like before PE Works, it was an unstructured Physical Education class and it was more of a recess time. With PE Works, there’s structure, there’s content, there’s integration with other subject areas. And I think it raises the expectations of the teachers and of the school leaders that this is an area as important as the other cluster areas.” - The work is supported and understood by the whole school because I think that some-- in essence, you can't teach PE if you don't have PE teacher. And you can't teach PE if you're not programming PE to take place at certain time or days of the week. But there are all these domino effects. The school may feel like there is not enough time to program it because we have to have math and we have to have English. So I think there needs to be an understanding among the school staff that this is a priority for the school. And the PE teacher really needs to be motivated and recognized for the value of the work that they bring to the school.” |
| **ADOPTION, Driver of Success 2:** Need appropriate, actionable indicators for auditing PE programs | |
| - “Our initiative was to focus on staffing, scheduling and programming, because that's what we thought we could influence. That's where we asked for money because that's what we're set up to do. But it not being under us to influence the schools’ physical environments remained a barrier.” - “I think one of the things that we might have struggled with was having too much data and not enough people to analyze the data and use the data. So I think we-- again, it comes back to asking smarter questions and less questions that are more strategic or having more statistical support to actually analyze that data and present it in a way that the program folks can use more readily.” | - “I would recommend focusing on data and fund what are the indicators that you actually would have resources to do anything about, right?” - “It was towards the beginning of our experience with PE Works. The needs assessment. That was one of the reasons I added the two additional certified PE teachers. To make sure we were in full compliance and all my students were getting the required minutes a week.” |
| **ADOPTION, Driver of Success 3**: Need an efficient data entry system and automated process for producing feedback reports | |
| - “We spent, I mean, an incredible amount of work making the SurveyGizmo [data entry tool] be as easy-to-do data entry as possible, which made a massive tool on the back end. Since creating that data entry process was an enormous amount of work. And making sure that it was accurate and clean and not glitchy.” - “The individuals creating the action plans -- that was just such an immense amount of work. And that's not to say a lack of effort. I think it's just how intense that process was. And we could have used more staff to do it. So using that data on individual schools faster and getting back the action plans and having those conversations with schools faster is probably something that could have been great. We did the best we absolutely could in that way. I think the other way is at the aggregate data that we really struggled with, was using the data more in real-time to pull the data into aggregates and provide stats back, for the district overall. The office could be like, “Here’s the trend." We really didn't have that capacity and could have used more statisticians to do that.” | N/A |
| **ADOPTION, Driver of Success 4:** Need appropriately qualified and passionate district-level personnel who are working collaboratively to work with schools | |
| - “There is a unique structure in that we have instructional team members to provide the instructional work in schools. So, if principals were asking about assessments, if principals were asking for support for new teachers, we people who could do both. And we also had people to help with the things that instructional specialist might not be good at, answering emails, helping program, some of the nitty-gritty things that are administrative that I think the set up then did allow for that to be a successful model because we had the people who are the bleeding heart PE people and then we had other folks who brought administrative skills to the table.” - “Just ongoing learning, communication, dialogue, within the team. And that took a lot more time and work than I anticipated. I think one of the big things I learned is that a team of this size, and with an initiative like this that's moving so quickly, if you think you need one meeting about it or one training on it, you probably need five meetings and five trainings to really make sure everyone is really hearing and understanding the same thing, is working through the discrepancies, the questions, the unexpected things that come up when you get out there in real life.” | - “I think for one, they really need to have some kind of knowledge base of schools in general. So that was always helpful because sometimes you have folks come to us that weren't necessarily school-based people. And then they have those that were former teachers, part of instructional staff. So those that had that educational background and experience could also be able to engage with an administrator in a different way than someone that comes through it with like a public health lens. So having that understanding of schools, coming to it with the idea of, "This is just for us to have a conversation on how to improve," People were really like they had to be friendly and humble and having an idea of how schools function and then also understanding some of the programmatic, sort of like what are the programs they can offer, having a baseline knowledge of regulations, programs, that's all helpful that would help the conversation more that than someone who didn't have some of that baseline knowledge.” |
| **IMPLEMENTATION, Driver of Success 1:** Need a personalized assessment of, and structured plan for, each school, which includes proactive coaching to improve PE | |
| - “This is most certainly a journey. It's not a destination. Right? I think that's important to note because once you have the needs assessment, once you have the summary report, once you have the action plan, you're not done. Right? That's the easy part. The actual work is the after. I think, intentional and systematic systems and structures in place and checkpoints. And I mean, it's the whole education leadership. It's the checkpoints, the progress monitoring, the feedback. All of that is actually really important.” - Every school is different, every school has a specific environment, facilities, and teachers, right, that comes with their own expertise and things that they're able to kind of do and are passionate about. So, because of PE Works, we were able to influx the school with not just the team but also supporting the teacher through training, but also providing equipment. Those that needed additional instructional support, there was a coach there available to help them through that process. So before, none of that existed.” | - “I mean, it's a great process. And if you have the capacity to do the supports, this is a great way to find out what your schools need. Instead of guessing, you're using collected data to really create meaningful things that are needed. Instead of just creating to create, you're creating with a purpose. So, I feel like these assessments, I can tell you, I still reference ours today. We haven't done them in two or three years and I still go back and look at all of mine to see where my school was, what we're doing now, and if we've made any great changes. So, again, really, they're great. They're a great way to learn the ins and outs of your school.” - “I think it also went to communicating to school communities that they were in this for the long term. They were very much saying, "We need to work together on this, but we're not expecting that you're going to go from zero to amazing in the course of one school year.” |
| **IMPLEMENTATION, Driver of Success 2:** Building strong district-school relationships is necessary for successful PEAFC implementation | |
| - “It's the relationships you build. You can tell a principal all you want about their mandates for PE and what is required by the state and what a PE program should look like and sound like and feel like-- a quality program that is. But until they actually believe you and until they actually trust you enough that you have their best interests at hand, that's when they start-- that's what makes it easy, easier. And there're just some principals that just won't, right? And that's okay. Then you have to kind of go, "Well, that's the mandate." And that's what-- it is what it is. But looking back for me, what made this school successful and move from point A to point B or point A to point D, it was a relationship that was built, and not just the initial needs assessment visit.” - “If we want to really cement our relevance and our importance in a school community, we have to approach it as, yes, we're coming in for PE, but we know what's happening in the school. We know what's happening in our school district. We know what the issues are. We're not coming in with blinders on, talking about PE. And that's the attitude we also need to take when we're having a conversation with principals about their PE program. We're not there to tell them, "You must." We're there to tell them, "Why can't you?" and "How can we help?" And I think the needs assessment is not a thing in of itself. It's what are the conversations?” | - “I found it useful having the conversation with [district-level personnel]. And then she would send me something in writing afterwards. Having the conversation with a summary of what we discussed and what the next step should be.” - “Well, what it did for me is, having been in the system for so long, I’m used to people over-promising. So it was good to actually have someone sit in my office, explain exactly how the process would work, what supports the school and teacher would receive. And then the follow-up. And the fact that they provided me true and accurate information was very helpful to build a trusting relationship, that it was just one of those, well here you are on your own type of things. That was definitely helpful because we are a big system, and it's very common for bureaucrats to over-promise and then leave you on your own. So it was nice to know that we were not on our own here.” - That frequent communication and not so much to where you feel like they’re hovering over you, but more so that they’re just there for support and questioning. Just being present in the early stages and then just being available for email, phone call, whenever, that was the most helpful thing for me. So just constant presence early, maybe the first one or two years, and then sort of hands off and transition out, show that there’s a bit of trust in what we are doing. |
| **MAINTENANCE, Driver of Success 1:** District-level personnel should remain available to schools to continue to support PE past the life of the program | |
| - “I think when we launched it, we originally thought that needs assessments would be ongoing. But it became pretty clear that we were never going to have the opportunity to do those kinds of needs assessments again. But if we could systematize the action plan, we wouldn't need to. There is a way to put it in place so that the community becomes the-- they're experiencing it. The students are experiencing it. What's the feedback loop? Because you really want the community to assess itself.” | - “So the actual paying of the teacher, over three years that disappeared. But PE Works continues to support the teachers and I don’t pay anything. I just like keeping the PE Works relationship because they help us with grants and other things and professional development and support. It’s like they’re part of the school, and I don’t pay for them. So, it’s great to have that.” |
| **MAINTENANCE, Driver of Success 2:** PEAFC should provide a long-term plan and structure for schools to successfully build and maintain a PE program | |
| - “We were building PE in schools and in the school system to be become embedded in the culture of the schools, the culture of school system, and to really be sustainable.” - “A lot of that professional development focused on these PE teachers being leaders in their schools. We worried that once PE teacher funding was out, these schools are going to have to scramble, now that they have to pay a quarter of this teacher's salary. So I was checking in, making sure that the schools were going to be contributing to the teachers' salary. And the feedback I got was that principals thought these teachers were amazing. They thought, "You are a leader in this school." They went in like dynamos. The family programs, weekend programs. They really look to the school community and their contribution. They really transformed the community. And that's really what solidified their existence in all of these schools.” | - “We were able to develop a wellness team. And it literally made PE something integrated and tangible for our children. It also turned into including families in it as well. So that it became an expectation at our school. It’s something we now do and do well.” - So PE is going to be something that is done over time. PE’s something that's always going to be there. That's something that's tied into instructional and graduation expectations. It's not a band-aid program. It's really having principals understand the instructional elements, and the concepts, and the standards of PE and how we can support student recovery, and that if we invest in PE programs, and other sort of student wellness programs, it's something that's going to be embedded in the school, it's not a one off thing, it's not somebody that comes in for six weeks and goes away. |
