# Supplementary material for: Implementation of elementary school physical education quantity and quality law through school district audit, feedback, and coaching
Source: Int J Behav Nutr Phys Act. 2023 Jun 29;20:77. doi: 10.1186/s12966-023-01479-1 (PMC10308623; doi:10.1186/s12966-023-01479-1)
Supplement: Supplementary file 2 — Additional file 2. [file 12966_2023_1479_MOESM2_ESM.pdf]

PE WORKS

A SCHOOL LEADER'S GUIDE TO QUALITY PHYSICAL EDUCATION

Think

Move

Achieve

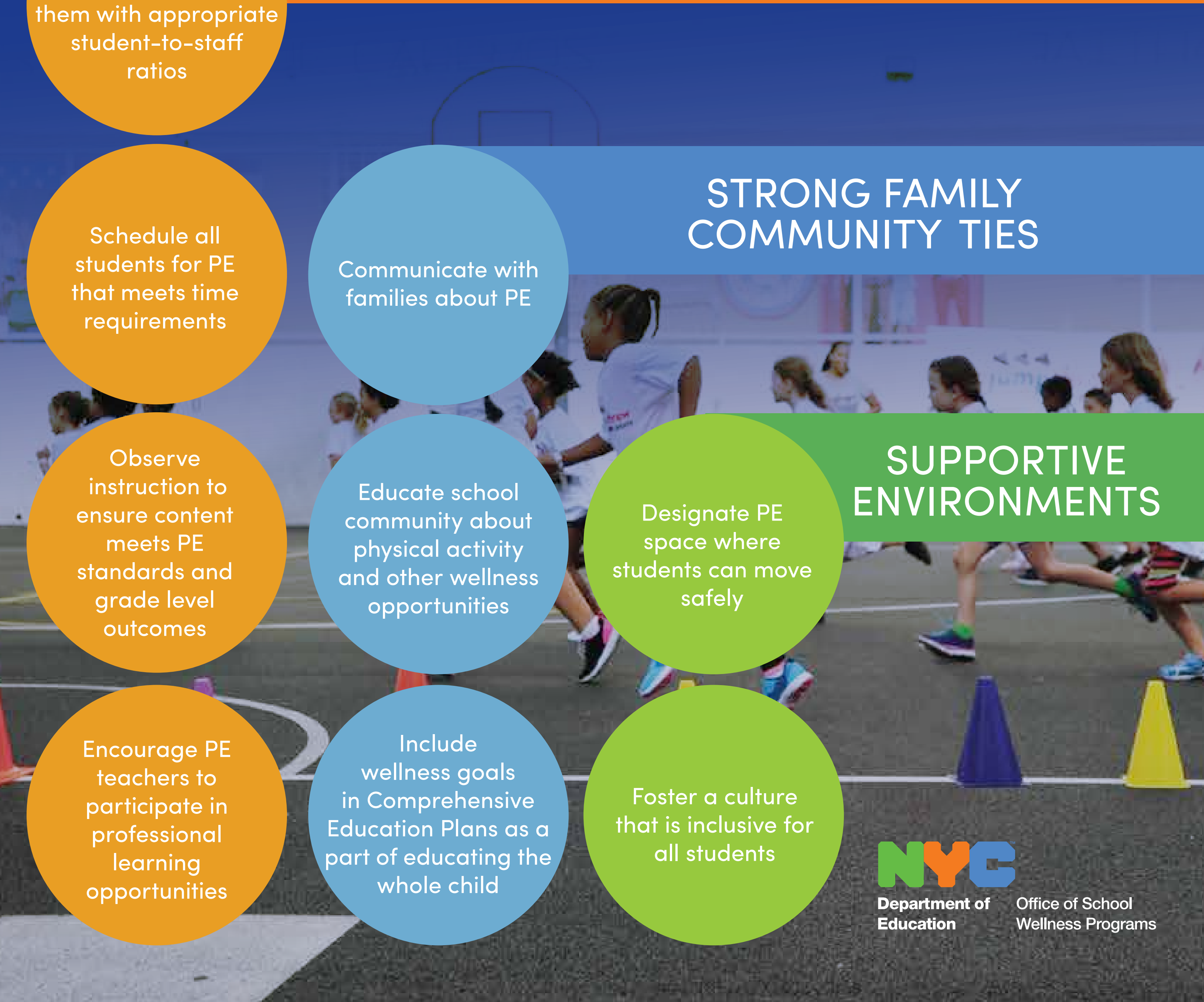

COLLABORATIVE TEACHERS AND RIGOROUS INSTRUCTION

- Hire certified PE teachers and assign them with appropriate student-to-staff ratios
- Schedule all students for PE that meets time requirements
- Observe instruction to ensure content meets PE standards and grade level outcomes
- Encourage PE teachers to participate in professional learning opportunities

STRONG FAMILY COMMUNITY TIES

- Communicate with families about PE
- Educate school community about physical activity and other wellness opportunities
- Include wellness goals in Comprehensive Education Plans as a part of educating the whole child

SUPPORTIVE ENVIRONMENTS

- Designate PE space where students can move safely
- Foster a culture that is inclusive for all students

NYC Department of Education

Office of School Wellness Programs

PE WORKS
A SCHOOL LEADER'S GUIDE TO QUALITY PHYSICAL EDUCATION

Think · Move · Achieve

SCHOOL NAME: SAMPLE SCHOOL

Here are the conditions you provide effectively to foster quality physical education (PE), and the ways we can help you sustain them:

Encourage learning
Your support for PE professional development contributes to quality instruction and keeps teachers engaged. We will continue to hone your PE staff's skills; develop leadership through professional learning plans and recognition; and, share information about free programs and other resources.

Communicate
Your parent communications ensure that students are prepared for PE, and that families can reinforce healthy habits outside of school. We will highlight what you do to inform families about when PE is scheduled, what students will learn, how students progress, and the benefits of PE and fitness.

Educate community
Your PE staff, CHAMPS coaches, and/or school wellness council members educate your school community by planning and sharing physical activity and wellness opportunities with students, families and staff. We will continue to inform you about programs, grants, partnerships, and events for your wellness champions to promote within your school community.

Foster inclusivity
Your PE environment encourages students to accept individual differences and develop inclusive behaviors. Staffmodel what students are expected to learn as part of PE. We will continue to offer your team professional development and resources on inclusivity as a core value for participation and learning in PE.

Here are the conditions in which we saw the need for improvement or need more information so that we can better collaborate towards improvement:

Staff
Certified PE teachers are the most important component of sustainable PE, so we will help you identify talented candidates who complement current staff. For elementary schools without a certified PE teacher, we will jumpstart hiring through PE Works funds and consult with you on how to establish appropriate staff-to-student ratios.

Schedule
PE scheduling improvements are key to ensuring all students are able to learn. To make sure your schedule meets time requirements for students equitably, and that it is reflected accurately in Central programming systems, we will provide you with guidance, and/or consult with you on how to improve scheduling. Elementary schools will receive free Move-to-Improve support to supplement daily PE minutes.

Observe
In year two of our plan, we will invite you to professional learning opportunities and provide you with guidance on observing PE to ensure content meets standards and grade level outcomes.

Include in CEP
Including wellness goals in your comprehensive education plan demonstrates a commitment to educating the whole child, and can show what your leadership team does to prioritize instructional requirements that enable students to learn and practice healthy habits. We will work with your PE staff and wellness champions to develop goals and engage your school community in meeting them.

Designate space
Improving the use of your space ensures that students can safely participate in quality PE. We will consult with you to help identify potential activity spaces inside and outside your building while sharing available tools, partnerships, and resources to help your PE staff maximize available spaces.
